# Un‐fractionated heparin counteracts the systemic inflammatory responses and multiple organ damages caused by endotoxaemia in sheep

**DOI:** 10.1002/vms3.720

**Published:** 2022-01-03

**Authors:** Hesam Ashareyoun, Aliasghar Chalmeh, Mehrdad Pourjafar

**Affiliations:** ^1^ Department of Clinical Sciences, School of Veterinary Medicine Shiraz University Shiraz Iran

**Keywords:** endotoxaemia, inflammation, multiple organ dysfunction, sheep, unfractionated heparin

## Abstract

**Background:**

Endotoxaemia is believed to be a major cause of mortality and there are several therapeutic regimens for the treatment of this situation.

**Objectives:**

The present experimental study was conducted to evaluate acute phase response, cardiovascular and hepatorenal damages following the treatment of Ovine experimental endotoxaemia model employing unfractionated heparin (UFH).

**Materials and Methods:**

Twenty clinically healthy 1‐year‐old fat‐tailed ewes were randomly divided into four equal groups, comprising UFH 200, UFH 400, Ctrl+ and Ctrl‐. Lipopolysaccharide (LPS) from *Escherichia coli* serotype O55:B5 at 0.4 μg/kg was administered intravenously to the ewes. UFH (at 200 and 400 IU/kg) was administrated to the UFH 200 and UFH 400 groups, respectively. All the ewes were evaluated clinically before and 1.5, 3, 4.5, 6 and 24 hours after LPS injection. Blood samplings were also performed at those hours. We measured serum concentrations of haptoglobin, interferon‐gamma, total antioxidant status, malondialdehyde, cardiac lactate dehydrogenase, cardiac troponin‐I, total bilirubin, alanine transaminase and creatinine. Serum concentrations of acute phase response, cardiovascular, hepatic and renal biomarkers and clinical parameters increased significantly following the induction of endotoxaemia in the groups receiving LPS.

**Results:**

The significantly lowest concentrations of these parameters at hours 4.5 and 6 among the treatment groups belonged to the UFH 400 sheep.

**Conclusions:**

UFH could act as an anti‐inflammatory mediator by decreasing inflammatory cytokines and acute phase proteins, modulating oxidative stress biomarkers and reducing multiple organ dysfunction following endotoxaemia in a dose–dependent manner. Furthermore, the anti‐inflammatory effects of UFH at 400 IU/kg were significantly higher than another dose. This research examined the effect of two doses of UFH and higher doses may have more anti‐inflammatory effects that require further studies.

## Graphical abstract

Endotoxemia induces acute phase response and multiple organ dysfunction in sheep. Unfractionated heparin at 400 IU/kg could alleviate inflammatory processes, oxidative stress and reduce serum concentration of multiple organ function biomarkers in endotoxin sheep.

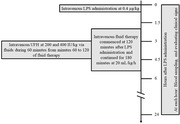

## 1 INTRODUCTION

Endotoxaemia is an acute inflammatory condition caused by lipopolysaccharide (LPS) of Gram‐negative bacteria in the bloodstream. This condition triggers a series of reactions in the host body called the acute phase response (APR) (Munford, 2016). APR reduces subsequent tissue damages by preventing further invasion of the pathogen, allowing the host body's haemostatic mechanisms to re‐establish their condition (Rodrigues et al., 2018). APR increases the production of a number of inflammatory mediators which are associated with local and systemic effects. Following APR, the production of inflammatory mediators, such as acute phase proteins and inflammatory cytokines, increases (Christoffersen et al., 2010). Therefore, by evaluating the circulating values of these parameters, APR could also be evaluated (Chalmeh et al., 2013). Oxidative stress is another event that occurs following APR, which could be assessed by measuring the circulating oxidative stress biomarkers (Rodrigues et al., 2018).

Increased production of inflammatory mediators following APR leads to secondary dysfunction of internal organs. Under these conditions, the components of the immune system, which are responsible for defending the host against invasive pathogens, could damage the host tissues and organs. This results in the failure of several organs, which is one of the main symptoms of endotoxaemia and is called multiple organ dysfunction (MOD) (Anderson et al., 2010). Accordingly, through evaluating the circulating biomarkers of each organ, such as liver, kidney and cardiovascular and respiratory system, it is possible to assess the health of the function of each of them (Chalmeh et al., 2014).

To prevent the exacerbation of acute inflammatory responses as well as disorders of internal organs, effective treatments should be taken into consideration for patients with endotoxaemia and inflammation. To date, several studies have been performed on different therapeutic regimens for patients with endotoxaemia in both human and animal models. Heparin is one of the drugs with the potential effects to treat inflammatory conditions (Poterucha et al., 2017). This drug could be classified into unfractionated heparin (UFH) and low molecular weight heparin (LMWH). UFH is the most widely used anti‐coagulant in clinical practice, which is recommended for the prevention of thromboembolism in septic patients; there is also information regarding its anti‐inflammatory effects (Li and Ma, 2017). There are several researches about anti‐inflammatory effects of UFH in asthma, cardiopulmonary bypass, inflammatory bowel diseases, acute coronary syndrome, cataract surgery and endotoxaemia at different aspects (Mousavi et al., 2015). Therefore, we hypothesized that UFH might be effective in reducing acute inflammatory reactions and reversing internal organ changes following endotoxaemia. Hence, the present study aimed to investigate the role of UFH in two different doses in the treatment of Ovine experimental endotoxaemia model by evaluating the inflammatory, oxidative stress, hepatic and renal and cardiovascular circulating biomarkers. The obtained results herein may also help to generalize the therapeutic effects of UFH to other inflammatory conditions.

## 2 MATERIALS AND METHODS

### 2.1 Animals

Twenty clinically healthy 1‐year‐old fat‐tailed ewes (50 ± 5 kg bodyweight) were randomly chosen for the project. Every sheep was given albendazole (15 mg/kg, orally; Dieverm 600, Razak Pharmaceutical Co.) and ivermectin (0.2 mg/kg, subcutaneously; Erfamectin 1 %; Erfan Pharmaceutical Co.) 4 weeks prior to the start of the experiments to control internal and external parasites. All of the ewes were kept in open‐shed barns with free access to water and shade. Alfalfa hay, corn silage, corn and barley were the main ingredients in the ration. They were then randomly allocated to one of the four experimental classes (n = 5): UFH 200, UFH 400, Ctrl+ and Ctrl‐.

### 2.2 Chemicals and drugs

The ewes were given 0.4 μg/kg of phenol extracted LPS from *Escherichia coli* serotype O55:B5 (Sigma–Aldrich; product No. L2880) as a bolus intravenous administration to induce endotoxaemia. In this study, each sheep was given only one dose of LPS, with no further administration permitted to prevent LPS tolerance phenomenon (Constable et al., 2017). This endotoxin was diluted in sterile phosphate‐buffered saline which was held at −80°C before endotoxaemia was induced. LPS was thawed and injected intravenously as mentioned below for each experiment. According to the experimental design, the UFH groups received intravenous injections of UFH (heparin sodium 5000, Alborz Pharmaceutical Co.). The intravenous fluid utilized in this study was 5% dextrose plus 0.45% sodium chloride (Shahid Ghazi Pharmaceutical Co.).

### 2.3 Induction and treatment of endotoxaemia

**FIGURE 1:**
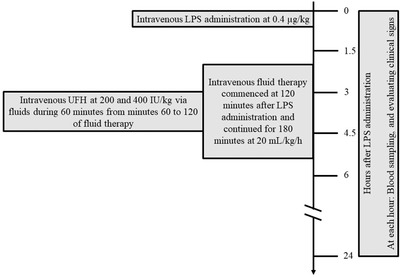
Schematic illustration of study design. At hour 0, experimental endotoxaemia induction was performed by intravenous LPS of *E. coli* serotype O55:B5 at 0.4 μg/kg in sheep. Fluid therapy was performed from 120 minutes after intravenous endotoxin administration for 180 minutes. Drugs were administrated via fluids at 180 minutes after intravenous endotoxin administration for 60 minutes. Blood samplings and evaluating clinical signs were performed at hours 0, 1.5, 3, 4.5, 6 and 24 after intravenous endotoxin administration

A Schematic illustration of the study design is presented in Figure 1. Clinical evaluations were performed on all the ewes before and 1.5, 3, 4.5, 6 and 24 hours after LPS injection. Rectal temperature, heart rate (HR) and respiratory rate (RR) were among the clinical parameters measured during the experiments. A 16‐gauge 5.1‐cm catheter was secured in the left jugular vein and used for blood samplings, endotoxin and drugs infusions and experimental procedures were commenced 1 h later, approximately, to prevent the effect of catheterization induced stress on the results. At hour 0, LPS was injected and intravenous fluid therapy began 2 hours later. In the related groups, UFH was infused via fluid between the 3rd and 4th hours, for 60 minutes. Following endotoxaemia induction, venous blood samples were taken at hours 0, 1.5, 3, 4.5, 6 and 24. Thawed LPS was diluted in 250 mL normal saline and injected at 10 mL/kg/h, intravenously. Fluid therapy with dextrose 5% plus sodium chloride 0.45% at 20 mL/kg/h was administered to all the experimental groups at 120 minutes following LPS injection. UFH and the fluid were injected at 180 minutes following the LPS administration. In the UFH 200 and UFH 400 groups, UFH was infused at 200 and 400 IU/kg BW, respectively. The positive control (Ctrl+) group received LPS, yet received no other treatments than intravenous fluid. The negative control group (Ctrl‐) received only intravenous fluids and no LPS and no drugs were administrated.

### 2.4 Blood sampling and serological assays

The blood samples were collected from all the ewes through the fixed catheter prior and 1.5, 3, 4.5, 6 and 24 hours after LPS injection in serum collection tubes. After the collections, sera were separated through centrifugation (for 10 min at 3000 × *g*) and stored at −20°C until assayed. The haemolysed specimens were not analysed by the laboratory.

Haptoglobin (Hp) was assayed with Ovine Hp ELISA kit (Bioassay Technology Laboratory Co.; sensitivity equal to 1.06 μg/mL; intra‐ and inter‐assay CV < 8% and 10%, respectively). Interferon‐gamma (IFN‐γ) was assayed with Ovine IFN‐γ ELISA kit (Bioassay Technology Laboratory Co., China; sensitivity equal to 2.12 ng/L; intra‐ and inter‐assay CV < 8 and 10%, respectively). Spectrophotometric method was employed to assay total antioxidant status (TAS) (Kiazist Life Science Co.; sensitivity equal to 20 nmol/mL; intra‐ and inter‐assay CV < 17 and 16%, respectively). We analysed malondialdehyde (MDA) employing colorimetric/fluorometric method (Kiazist Life Science Co.; sensitivity equal to 10 μM; intra‐ and inter‐assay CV < 15 and 19%, respectively). We also measured the values of serum cardiac lactate dehydrogenase (LDH) with Integra 800 autoanalyzer (Roche‐Cobes, Switzerland). The levels of serum cardiac troponin‐I (cTnI) were determined employing Ovine cTnI ELISA kit (Bioassay Technology Laboratory Co.; sensitivity equal to 2.34 ng/L; intra‐ and inter‐assay CV < 8 and 10%, respectively). Total bilirubin was analysed using the 2,4dichloroaniline photometric method (Pars Azmoon). The values of serum alanine transaminase (ALT) were measured with an Integra 800 autoanalyzer (Roche‐Cobes). To measure serum creatinine, we utilized Pars Azmoon Commercial kits.

### 2.5 Statistical analyses

The data were expressed as mean and pooled standard error of mean (SEM). Statistical analyses were conducted using the one‐way ANOVA with the Tukey post‐hoc test to compare the mean concentrations of different serological factors and clinical parameters within similar hours among different experimental groups. The trend alterations of all the studied parameters were evaluated employing the repeated measures ANOVA. The effects of group, time and their interactions on the changes of each parameter were also analysed. All the statistical analyses were conducted using the SPSS software (SPSS for Windows, version 22), and the significance level was set at *p* < 0.05.

## 3 RESULTS

After inducing endotoxaemia, serum concentrations of Hp increased significantly in the groups receiving endotoxin and the rising trend continued until 6 hours in the Ctrl+ group (Table 1; *p* < 0.05). The Hps in the Ctrl‐ group were significantly lower than those in the other studied groups at all the hours (except hour 0). After beginning the drug administrations, the levels of Hp in the treatment groups (at hour 4.5) decreased and were substantially lower than those in the Ctrl+ group (Table 1; *p* < 0.05). The UFH 400 receiving sheep had the lowest significant concentrations of Hp at hours 4.5, 6 and 24. Following intravenous endotoxin infusion, serum levels of IFN‐γ significantly increased in all the ewes (Table 1; *p* < 0.05). Following the administration of UFH to the related groups, their levels in the UFH 200 and UFH 400 significantly decreased. At hours 4.5, 6 and 24, the lowest significant concentrations of IFN‐γ belonged to the UFH 400.

**TABLE 1:** Alterations (Mean ± pooled SEM) of circulating acute phase protein (Hp), inflammatory cytokine (IFN‐γ) and antioxidant biomarkers (TAS and MDA) following experimental endotoxaemia induction by intravenous LPS of *E. coli* serotype O55:B5 at 0.4 μg/kg in sheep and its treatment by intravenous UFH at 200 and 400 IU/kg

Parameters	Groups	Hours						Pooled SEM	*p*‐value*		
		0	1.5	3	4.5	6	24		Group	Time	Group × time
Hp (g/dL)	Ctrl+	0.09^a^	0.38^a^	0.52^a^	0.64^a^	0.62^a^	0.52^a^	0.02	<0.001	<0.001	<0.001
	Ctrl‐	0.09^a^	0.1^b^	0.12^b^	0.15^b^	0.13^b^	0.09^b^	0.01			
	UFH 200	0.09^a^	0.4^a^	0.52^a^	0.38^c^	0.26^b^	0.09^b^	0.02			
	UFH 400	0.09^a^	0.42^a^	0.56^a^	0.32^c^	0.2^b^	0.08^b^	0.01			
IFN‐γ (pg/dL)	Ctrl+	26.97^a,b,c^	49.03^a^	64.98^a^	77.27^a^	88.56^a^	83.68^a^	0.54	<0.001	<0.001	<0.001
	Ctrl‐	28.53^a^	30.62^b^	31.67^b^	32.72^b^	31.53^b^	28.3^b^	0.36			
	UFH 200	27.52^b^	51.45^a^	68.89^a^	55.86^c^	40.76^c^	29.01^b^	0.33			
	UFH 400	25.21^c^	48.94^a^	64.97^a^	44.44^d^	29.22^b^	28.68^b^	0.24			
TAS (mmol/L)	Ctrl+	2.19^a^	1.1^a^	0.89^a^	0.62^a^	0.44^a^	0.3^a^	0.02	<0.001	<0.001	<0.001
	Ctrl‐	2.22^a^	2.3^b^	2.46^b^	2.45^b^	2.45^b^	2.25^b^	0.04			
	UFH 200	2.29^a^	1.2^a^	0.99^a^	0.79^a^	1.68^c^	2.32^b^	0.06			
	UFH 400	2.35^a^	1.26^a^	1.05^a^	0.85^a^	1.97^d^	2.38^b^	0.09			
MDA (mmol/L)	Ctrl+	0.61^a^	1.38^a^	1.92^a^	2.3^a^	2.63^a^	2.47^a^	0.03	<0.001	<0.001	<0.001
	Ctrl‐	0.63^a^	0.68^b^	0.66^b^	0.7^b^	0.65^b^	0.69^b^	0.02			
	UFH 200	0.63^a^	1.37^a^	1.85^a^	1.2^c^	0.83^c^	0.68^b^	0.03			
	UFH 400	0.61^a^	1.31^a^	1.79^a^	0.94^d^	0.57^b^	0.57^c^	0.02			

Ctrl+, positive control; Ctrl‐, negative control; UFH 200, unfractionated heparin at 200 IU/kg; UFH 400, unfractionated heparin at 400 IU/kg; Hp, haptoglobin; IFN‐γ, interferon‐gamma; TAS, total antioxidant status; MDA, malondialdehyde.

Different letters indicate significant differences among different groups at each study time about each parameter (*p* < 0.05).

*Effect of group, time and group × time following induction and treatment of endotoxaemia by different drugs about each studied parameter. The significance level is lesser than 0.05.

Following the induction of endotoxaemia, serum activity of TAS levels decreased significantly in all the endotoxin‐receiving sheep (Table 1; *p* < 0.05). This decrease persisted in the Ctrl+ group until hour 6, but it increased in the UFH groups after the drugs were administered. The UFH 400 group had significantly higher serum levels of TAS at hours 4.5, 6 and 24 compared to the other endotoxin‐receiving groups. Following endotoxin injection, serum MDA levels increased significantly in all the endotoxic ewes up to hour 3. At hours 4.5, 6 and 24, serum MDA concentrations in the UFH 400 group were significantly lower than those in the UFH 200 groups (Table 1; *p* < 0.05).

After inducing endotoxaemia, serum concentrations of cTnI and LDH increased significantly in the endotoxin‐receiving groups. This rise in the Ctrl+ group lasted until hour 6 (Table 2; *p* < 0.05). The serum concentrations of cTnI and LDH in the Ctrl‐ group were significantly lower than those in the other studied groups at all the hours (except hour 0). The levels of cTnI and LDH in the treatment groups decreased after the beginning of drug administrations (at hour 4.5), which were significantly lower than those in the Ctrl+ group (Table 2; *p* < 0.05). At hours 4.5, 6 and 24, the UFH 400‐receiving sheep had the least significant concentrations of cTnI, and LDH.

**TABLE 2:** Alterations (Mean ± pooled SEM) of circulating cardiovascular (cTnI and LDH), hepatic (ALT and T. Bil.) and renal (creatinine) biomarkers following experimental endotoxaemia induction by intravenous LPS of *E. coli* serotype O55:B5 at 0.4 μg/kg in sheep and its treatment by intravenous UFH at 200 and 400 IU/kg

Parameters	Groups	Hours						Pooled SEM	*p*‐value*		
		0	1.5	3	4.5	6	24		Group	Time	Group × time
cTnI (ng/mL)	Ctrl+	0.18^a^	0.47^a^	0.57^a^	0.62^a^	0.69^a^	0.48^a^	0.01	<0.001	<0.001	<0.001
	Ctrl‐	0.18^a^	0.19^b^	0.19^b^	0.19^b^	0.19^b^	0.18^b^	0.01			
	UFH 200	0.19^a^	0.47^a^	0.57^a^	0.51^c^	0.41^c^	0.33^c^	0.01			
	UFH 400	0.19^a^	0.48^a^	0.58^a^	0.41^d^	0.32^d^	0.26^d^	0.01			
LDH (IU/L)	Ctrl+	854.04^a^	1537.27^a^	1844.72^a^	2029.2^a^	2252.41^a^	1126.2^a^	20	<0.001	<0.001	<0.001
	Ctrl‐	846.04^a^	849.65^b^	844.04^b^	840.17^b^	852.63^b^	851.8^b^	8.35			
	UFH 200	855.26^a^	1539.47^a^	1847.37^a^	1477.89^c^	1182.31^c^	945.85^c^	9.98			
	UFH 400	834.1^a^	1501.39^a^	1801.67^a^	1080.99^d^	918.84^b^	872.9^b^	12.52			
ALT (U/L)	Ctrl+	20.25^a^	36.45^a^	51.02^a^	61.23^a^	67.36^a^	47.15^a^	0.56	<0.001	<0.001	<0.001
	Ctrl‐	20.73^a^	20.24^b^	20.33^b^	20.57^b^	20.17^b^	20.25^b^	0.32			
	UFH 200	20.77^a^	37.39^a^	52.35^a^	41.88^c^	33.50^c^	28.47^c^	0.48			
	UFH 400	20.16^a^	36.28^a^	50.79^a^	35.55^d^	28.44^d^	22.75^d^	0.49			
T. Bil. (μmol/L)	Ctrl+	3.44^a^	4.12^a^	4.95^a^	5.44^a^	5.98^a^	4.79^a^	0.06	<0.001	<0.001	<0.001
	Ctrl‐	3.4^a^	3.41^b^	3.45^b^	3.45^b^	3.46^b^	3.48^b^	0.07			
	UFH 200	3.51^a^	4.21^a^	5.04^a^	4.94^c^	4.84^c^	4.74^a^	0.09			
	UFH 400	3.45^a^	4.14^a^	4.96^a^	4.36^d^	4.27^d^	3.85^c^	0.07			
Creatinine (mg/dL)	Ctrl+	1.04^a^	1.34^a^	1.48^a^	1.33^a^	1.26^a^	1.14^a^	0.01	<0.001	<0.001	<0.001
	Ctrl‐	1.03^a^	1.03^b^	1.03^b^	1.03^b^	1.03^b^	1.03^b^	0.01			
	UFH 200	1.03^a^	1.34^a^	1.47^a^	1.18^c^	1.06^c^	1.04^b^	0.01			
	UFH 400	1.02^a^	1.32^a^	1.46^a^	1.11^d^	1.02^b^	1.03^b^	0.01			

Ctrl+, positive control; Ctrl‐, negative control; UFH 200, unfractionated heparin at 200 IU/kg; UFH 400, unfractionated heparin at 400 IU/kg; cTnI, cardiac troponin I; LDH, lactate dehydrogenase; ALT, alanine transaminase; T. Bil., total bilirubin.

Different letters indicate significant differences among different groups at each study time about each parameter (*p* < 0.05).

*Effect of group, time and group × time following induction and treatment of endotoxaemia by different drugs about each studied parameter. The significance level is lesser than 0.05.

In all the endotoxic animals, serum levels of hepatic biomarkers (ALT and total bilirubin) increased significantly after intravenous endotoxin infusion (Table 2; *p* < 0.05). UFH significantly reduced the levels of these hepatic biomarkers. At hours 4.5, 6 and 24, the least significant concentrations of ALT and total bilirubin were detected in the UFH 400. After endotoxin injection, serum renal biomarker (creatinine) levels of all the studied groups increased substantially and all the endotoxic sheep's levels increased up to hour 3. At hours 4.5, 6 and 24, serum creatinine concentrations in the UFH 400 group were significantly lower than those in the UFH 200 (Table 2; *p* < 0.05).

Following the induction of endotoxaemia, HR and RR and rectal temperature increased significantly. The increasing pattern of the Ctrl+ group persisted until hour 4.5 (Table 3; *p* < 0.05). These clinical parameters were significantly lower in the Ctrl‐ group than in the other studied groups at all the hours (except for hour 0). The quantities of HR, RR and rectal temperature in the treatment groups decreased after beginning drug administration (at hour 4.5), which were significantly lower compared with those in the Ctrl+ group (Table 3; *p* < 0.05). HR, RR and rectal temperature were found to be significantly lower in the UFH 400 sheep at hours 4.5 and 6. At hour 24, there were no significant differences concerning the amounts of these parameters among the studied groups (*p* > 0.05).

**TABLE 3:** Alterations (Mean ± Pooled SEM) of clinical signs (HR, RR and rectal temp.) following experimental endotoxaemia induction by intravenous LPS of *E. coli* serotype O55:B5 at 0.4 μg/kg in sheep and its treatment by intravenous UFH at 200 and 400 IU/kg

Parameters	Groups	Hours						Pooled SEM	*p*‐value*		
		0	1.5	3	4.5	6	24		Group	Time	Group × time
HR (beats/min)	Ctrl+	74^a^	144^a^	149^a^	147.8^a^	134.4^a^	77.8^a^	1.25	<0.001	<0.001	<0.001
	Ctrl‐	75^a^	83.8^b^	94.8^b^	92.4^b^	79.4^b^	82.4^a^	1.29			
	UFH 200	75.4^a^	145.4^a^	148.4^a^	127.4^c^	106.4^c^	78^a^	1.1			
	UFH 400	77.6^a^	147.6^a^	145.2^a^	110.2^d^	89.2^d^	78.4^a^	1.26			
RR (breath/min)	Ctrl+	23.8^a^	55.8^a^	63.8^a^	71.8^a^	66.8^a^	24.8^a^	0.54	<0.001	<0.001	<0.001
	Ctrl‐	24.8^a^	24.4^b^	33.4^b^	32.2^b^	25^b^	24.8^a^	0.58			
	UFH 200	25.2^a^	57.2^a^	65.2^a^	40.2^c^	32.2^c^	23.4^a^	0.43			
	UFH 400	24^a^	56^a^	64^a^	34^b^	26^b^	24.6^a^	0.54			
Rectal Temp. (°C)	Ctrl+	39.18^a^	40.58^a^	40.48^a^	40.26^a^	40.08^a^	39.2^a^	0.03	<0.001	<0.001	<0.001
	Ctrl‐	39.16^a^	39.14^b^	39.18^b^	39.22^b^	39.2^b^	39.2^a^	0.03			
	UFH 200	39.14^a^	40.54^a^	40.44^a^	40.04^c^	39.74^c^	39.24^a^	0.02			
	UFH 400	39.22^a^	40.62^a^	40.52^a^	39.72^d^	39.42^d^	39.22^a^	0.03			

Ctrl+, positive control; Ctrl‐, negative control; UFH 200, unfractionated heparin at 200 IU/kg; UFH 400, unfractionated heparin at 400 IU/kg; HR, heart rate; RR, respiratory rate; Rectal Temp., rectal temperature.

^a,b^Anti‐inflammatory effects of heparin and its derivatives: a systematic review.

Different letters indicate significant differences among different groups at each study time about each parameter (*p *< 0.05).

*Effect of group, time and group × time following induction and treatment of endotoxaemia by different drugs about each studied parameter. The significance level is lesser than 0.05.

## 4 DISCUSSION

Endotoxaemia is known to be a leading cause of morbidity around the world. As a result, implementing adequate treatment methods that are efficient, reliable, easy and inexpensive is critical in treating this situation. These therapies should have the fewest possible side‐effects and be widely available (Mousavi et al., 2015). Therefore, the current research sought to investigate the anti‐inflammatory effects of UFH in an Ovine experimental endotoxaemia model by analysing the APR and MOD. Several animal and human studies have suggested that heparin not only effectively prevents the coagulation mechanism in endotoxaemia, but also causes a variety of anti‐inflammatory responses against infection. Human endotoxaemia experimental models have indicated that heparin can treat endotoxaemia by inducing coagulation activities (Jaimes et al., 2006; Li et al., 2009; Li et al., 2013; Miranda et al., 2014; Schiffer et al., 2002; Wyns et al., 2015; Zhao et al., 2017). However, in human models, the inhibition of inflammatory pathways following endotoxaemia has not been as clearly demonstrated as in animal models. The current research explored the anti‐inflammatory effects of UFH in an Ovine endotoxaemia model, focusing on inflammatory, oxidative stress, cardiovascular, hepatic, renal and clinical indices to generalize it to humans and other inflammatory conditions. In this research, we compared the efficacy of two different doses of UFH (200 and 400 IU/kg) in endotoxic sheep. The current study found that UFH at 400 IU/kg was effective in reducing inflammation and organ dysfunction in sheep following endotoxaemia.

Heparin is the most commonly available and least expensive anti‐coagulant utilized to treat DIC. Haneberg et al. (1983) conducted the first randomized human clinical trial of heparin in 1983, studying 26 babies and children with acute meningococcal sepsis. Eleven patients were given intravenous heparin “as soon as possible after admission” and on a continuous basis for 2 days. Standard care was given to only 15 children. There were two deaths in each group, and the clinical course of the patients who survived did not indicate any major changes. This research investigated admission duration, clinical seriousness, comorbidities, concomitant treatments and other potentially important differences between the groups (Haneberg et al., 1983).

Several animal and human trials have been conducted to investigate the possible benefits of heparin in the treatment of bacterial infections. Heparin may increase cardiac output and oxygen delivery (Redmond et al., 1993). They used *E. coli* endotoxin on 14 sheep continuously for 24 hours. Seven sheep were given a fixed dose of 5000 units of heparin every 4 hours after endotoxin injection while seven other sheep served as controls. Heparinized animals demonstrated a three‐phase cardiovascular response with a rise in the cardiac index and a decrease in systemic vascular resistance in the first 2 hours, followed by a return to baseline in about 4 hours. The cardiac index improved and systemic vascular resistance decreased dramatically in the final stage (8 to 24 hours).

Schiffer et al. (2002) investigated the anti‐inflammatory effects of UFH and hirudin on sheep after endotoxaemia induction. They randomly assigned 22 sheep to one of their three treatment groups: (a) normal saline, (b) continuous infusion of conventional un‐fractioned bovine lung heparin and (c) continuous injection of recombinant hirudin. *Escherichia coli* endotoxin was administered after a 6‐hour baseline evaluation period. Endotoxin injections killed all the sheep in the control and hirudin groups, but four of the seven animals which were given continuous heparin injections lived to the end of the trial. The animals in the control group died between 6 and 44 hours after the start of endotoxin administration while the animals in the hirudin group died between 8 and 30 hours. Between 48 and 56 hours after the beginning of endotoxin administration, three sheep died in the heparin group. When compared to the other two treatment animals, the difference in survival rate was statistically significant in this group (Schiffer et al., 2002).

Li et al. (2020) studied the signalling mechanisms that may be involved in UFH's anti‐inflammatory effects on LPS‐stimulated human pulmonary microvascular endothelial cells. They discovered that UFH clearly inhibited LPS‐stimulated IL‐6 and IL‐8 production. Furthermore, UFH inhibited the phosphorylation of IκB‐α, ERK1/2, JNK, p38 MAPK and STAT3 by LPS. UFH also inhibited LPS‐induced nuclear translocation of NFκ‐B. More importantly, siRNA targeting IκB‐α induced a more evident inflammatory response. In IκB‐α silencing cells, UFH inhibited cytokine synthesis and phosphorylation of various signalling pathways. Their findings demonstrated that UFH exerts anti‐inflammatory effects through various signalling pathways (Li et al., 2020).

Derhaschnig et al. (2003) evaluated UFH's anti‐inflammatory properties in human endotoxaemia. Following endotoxaemia, they found that UFH had little effect on cytokine production and endothelial cell activation. UFH also decreased l‐selectin downregulation and lymphocytopenia (Derhaschnig et al., 2003). Heparin has been shown in several studies to reduce the inflammatory response following cardiopulmonary bypass. The use of heparin‐treated surfaces in cardiopulmonary bypass circuits has been shown to reduce leukocyte activation and complement cascade activation (Ludwig, 2009). As a result, the need for inotropic support, post‐operative mechanical ventilation time and incidence of acute lung injury, on top of the length of hospital stay, decrease, reflecting heparin's beneficial impact in cardiopulmonary bypass circuits (Redmond et al., 1993; Svenmarker et al., 1997). In these trials, the heparin‐coated circuit significantly reduced the levels of cytokines, such as TNF‐α, complement complex, neutrophils and elastase, when compared to the non‐heparin‐coated circuit. The current study found that UFH at 400 IU/kg significantly decreased the inflammatory process and combated MOD after endotoxaemia induction in the studied sheep (Tables 1, 2, 3; *p* < 0.05). According to our results, UFH at 400 IU/kg had anti‐inflammatory properties superior to 200 IU/kg.

IFN‐γ is the only member of class II IFNs and is important for both innate and adaptive immunity against viral and intracellular bacterial infections. The role of IFN‐γ in the immune system is owing to its ability to directly inhibit virus replication as well as its immune‐stimulating and regulating effects (Parameswaran & Patial, 2010; Schroder et al., 2004). IFN‐γ reduced in the UFH 200 and UFH 400 after the drugs were administered to these groups. Following the therapy, the concentrations of this cytokine in the UFH 400 were significantly lower than those in the UFH 200 (Table 1; *p* < 0.05). Since IFN‐γ is a marker of inflammation and APR, its substantial reduction following UFH administration at 400 IU/kg, when compared to other drug‐receiving ewes, may suggest that UFH has the potential to reduce inflammation following endotoxaemia in this Ovine endotoxaemia model.

Hp is an acute phase protein that has been proposed as a stress biomarker in animals (Eckersall and Bell, 2010). Hp is synthesized in the liver and is a key acute phase protein in several animal species. The amount of Hp in the bloodstream of ruminants is usually negligible, but it increases more than 100‐fold once the immune system is stimulated (Eckersall and Bell, 2010; George & Sack, 2018). Several studies have highlighted the significance of Hp as a useful para‐clinical parameter for assessing the occurrence and severity of inflammatory diseases in sheep (Chalmeh et al., 2014). Serum Hp determination has shown that this protein can be useful in the diagnosis of infection and inflammatory conditions. According to Chalmeh et al. (2013), serum concentrations of Hp increased significantly after inducing endotoxaemia in sheep. UFH decreased the circulating levels of Hp following the start of the treatments in the current study; however, UFH at 400 IU/kg significantly reduced the concentrations of this protein as compared to the UFH 200 group (Table 1; *p* < 0.05). This may explain why heparin has anti‐inflammatory properties that help to reduce the inflammation caused by endotoxaemia.

The pro‐ and antioxidants could be used to measure oxidative stress. MDA is a pro‐oxidant derived from lipid peroxidation; however, it is a weak indicator of oxidative damage (Tsikas, 2017). Nonetheless, we employed this parameter to assess lipid peroxidation. Endogenous antioxidants are a network of substances in the body that can deal with an oxidative assault. Antioxidant enzymes are a form of endogenous antioxidant. Since other forms of antioxidants are not taken into account, assessing the enzymes alone cannot decide the status of antioxidants throughout the body (Valko et al., 2007). As a result, by assessing TAS, it would be possible to determine the combined action of all the antioxidants present in the target samples (Erel, 2004). In the current research, circulating levels of TAS significantly reduced after endotoxin infusion with the lowest levels observed 24 hours after endotoxaemia induction in the Ctrl+ ewes (Table 1; *p* < 0.05). These findings indicated that the major evolving patterns of TAS are consumptive during APR after endotoxaemia induction. Following the therapies, TAS levels increased and at hour 24, these levels were similar to those in the baseline in the UFH 200 and UFH 400 groups. The current study found that the serum concentration of oxidative stress biomarkers in the UFH 400 ewes was not significantly different from that in the Ctrl‐ sheep at hour 24 (Table 1; *p* > 0.05). This result could be explained through UFH's efficacy in decreasing reactive oxygen species pathways; thus, modulating oxidative stress could be done more potently by UFH at 400 IU/kg than 200 IU/kg.

Examining circulating cardiovascular biomarkers may also provide useful information about the cardiovascular health. Several studies have reported that once myocardium and endothelium are impaired, circulating levels of cTnI and enzymes like LDH augment (Aldous, 2013). Endotoxaemia can affect physiological cardiovascular functions; accordingly, cardiovascular isoenzymes and biomarkers alter (Chalmeh et al., 2014). As a result, these diagnostic biomarkers are useful parameters in the early detection of cardiovascular complications caused by ischaemia, injury, or inflammation. The literature describes the changes in cardiac injury biomarkers following endotoxaemia induction in farm animals (Chalmeh et al., 2014; Peek et al., 2008). Serum concentrations of cardiovascular biomarkers increased rapidly and significantly at hour 1.5 after endotoxaemia induction (Table 2; *p* < 0.05) and remained elevated in the Ctrl+ ewes until hour 24. The emergence of cTnIs as the gold standard, as well as responsive and precise biochemical markers of myocardial injuries, has helped the diagnosis and management of myocardial injuries over the recent years (Aldous, 2013). The rise in cTnI immediately after LPS administration in the current study could indicate myocardial injuries during endotoxaemia. Increased cTnI have been linked to a variety of animal cardiac disorders. Serum cardiac LDH levels significantly increased 1.5 hours after endotoxin infusion in all the endotoxin‐receiving sheep (Table 2; *p* < 0.05). In the Ctrl+ animals, high concentrations of these enzymes were observed at all the hours after endotoxaemia induction and remained elevated up to hour 24 after endotoxin administration. LDH is a cytoplasmic enzyme found in the heart, skeletal muscle, liver, kidney and red blood cells. This enzyme is a marker of increased cellular damage and its increased activity is a result of its increased release from damaged cells, as well as a reflection of metabolic changes in inflamed tissues, specifically the heart (Aldous, 2013). Damage to the skeletal or heart musculature causes a significant rise in serum LDH levels since the majority of the vessels in the body can be thought of as a sufficient reservoir of enzymes liable to be released and identified during pathological situations. As a result, any damage to the vasculature could trigger enzyme leakage, making it a valuable tool in the early detection of pathological conditions (Pourjafar et al., 2013).

The findings in the current study indicated that circulating cardiovascular biomarkers significantly increased after endotoxaemia induction in the sheep receiving endotoxin, reflecting the severe cardiovascular damage in this acute inflammatory state. UFH reduced cardiovascular biomarkers and UFH at 400 IU/kg was significantly more effective than UFH at 200 IU/kg. Hence, it is possible to state that UFH is of anti‐inflammatory effects on reducing cardiovascular damage and dysfunction in endotoxic sheep.

Liver plays an important role in protective responses to scavenge bacteria and the development of inflammatory mediators during the endotoxaemia (Yao et al., 2013). Endotoxaemia‐related liver dysfunction has been historically thought to be a late feature of serious illness, manifesting as jaundice and hyperbilirubinaemia (Lida et al., 2009). Recent studies, however, have identified liver dysfunction as an early event in endotoxaemia (Marshall, 2012). As a result, it is critical to comprehend the pathophysiological changes leading to endotoxaemia‐related liver dysfunction, which has been characterized as a combination of cellular injury and increased inflammation. Endotoxaemia may cause damage to hepatocytes. This damage is caused by hypoperfusion in the presence of hypovolaemia and insufficient cardiac performance and could be repaired quickly with supportive therapy (Rivera et al., 2003). This injury is distinguished through the leak of transaminase enzymes, which represents acute cellular and mitochondrial damages. According to the literature, ALT is rapidly elevated following an episode of hypotension or shock (Kobashi et al., 2013). When the underlying path was reversed, ALT levels dropped dramatically in a matter of days. Septic shock combined with hypoxic hepatitis can lead to fulminant hepatic failure, which often results in disseminated intravascular coagulation and bleeding. Lactate and amino acid clearance, on top of protein synthesis, reduces after hepatic injuries caused by endotoxaemia. Gluconeogenesis and glycogenolysis are also reduced and hypoglycaemia may occur. In endotoxaemia, jaundice is much more prevalent than hypoxic hepatitis. Jaundice is usually a late complication of serious endotoxaemia. However, even in the absence of fever or leukocytosis, jaundice may occur at an early stage of endotoxaemia (Famularo et al., 2003); hence, serum bilirubin is the most commonly used biomarker for detecting hepatic dysfunction during sepsis (Patel et al., 2013). Bilirubin levels, on the other hand, lack precision in reflecting the broad range of liver dysfunction and distinguishing an acute reaction from a pre‐existing organ chronic disease. The obtained results herein revealed that serum levels of ALT and total bilirubin increased significantly after endotoxaemia induction (Table 2; *p* < 0.05) and this pattern persisted to hour 24 in the Ctrl+ ewes. Thus, endotoxaemia can cause hepatocyte damage, resulting in an increase in ALT and total bilirubin. Furthermore, owing to the anti‐inflammatory properties of the used drugs in this research, UFH prevented further liver damage. In the studied endotoxic sheep, the concentrations of circulating ALT and total bilirubin decreased greatly. According to the current findings, UFH at 400 IU/kg had a greater potency to reduce the concentrations of ALT, and total bilirubin, thereby preventing further damage to the liver.

Creatinine, a waste product of phosphocreatine catabolism, is primarily filtered by the kidney though a limited amount is deliberately secreted. While there is some tubular reabsorption of creatinine, this is offset by nearly equal amounts of tubular secretion. Any increase in creatinine levels in the blood is related to excretion and, thus, indicates kidney dysfunction. However, in case of extreme renal impairment, creatinine clearance may be overestimated due to active creatinine secretion, which accounts for a greater fraction of overall creatinine cleared. Higher levels of it than normal could indicate dehydration (Uchino, 2010). Understanding the effects of endotoxaemia on renal blood flow, glomerular filtration rate, renal vasculature and tubular function is critical for identifying potential intervention targets to reduce the complications of endotoxaemia‐induced acute renal failure (Wang et al., 2010). In the current study, all the endotoxic sheep had significantly higher circulating creatinine levels after endotoxin infusion (Table 2; *p* < 0.05). The creatinine rise in this work was most likely due to endotoxin damage to renal tissue and its inability to expel creatinine from the body. Since all the animals in our study received intravenous fluids, an increase in blood creatinine after endotoxaemia could not be attributed to dehydration in the endotoxin‐receiving ewes. 4.5 and 6 hours after endotoxaemia induction, serum creatinine concentrations in the UFH 400 group were significantly lower than those in the UFH 200 (Table 2; *p* < 0.05). As a result, UFH at 400 IU/kg was able to minimize renal failures following endotoxaemia in the sheep and this capacity may be on account of its anti‐inflammatory properties.

The body temperature increases after endotoxin administration and it is a right pyrogenic reaction (Centanni, 1984). Small doses of endotoxin induce a monophasic fever response in common laboratory animals whereas moderate to large doses can induce a biphasic fever response. The initial fever response, which is caused by a direct impact on the thermoregulatory centre in the hypothalamus, is thought to have a 1‐hour latency period. If a second high occurs, it appears 4 hours after the endotoxin is administered (Coskun et al., 2020). Endotoxaemia causes fever and in the current study, the rectal temperature of the endotoxic sheep increased significantly after endotoxin infusion (Table 3; *p* < 0.05). Following drug administrations, this clinical parameter decreased significantly in the treatment groups. At hours 4.5 and 6, the rectal temperature in the UFH 400 was significantly lower than that in the UFH 200. Rectal temperatures at hour 24 after endotoxin infusion did not vary significantly between the groups. Based on the findings, we could conclude that UFH at 400 IU/kg was more potent in reducing inflammatory and pyrogenic processes than UFH at 200 IU/kg.

HR fluctuations are a physiological phenomenon primarily regulated by the autonomic nervous system. Therefore, HR monitoring is a non‐invasive method that could be utilized to investigate the complex equilibrium of sympathetic and parasympathetic activities. It has been demonstrated that HR analysis is a sensitive method for studying the autonomic nervous system in animals and detecting discrete changes in sympathetic‐parasympathetic balance, particularly, vagal function. The inflammatory response and the autonomic nervous system are inextricably related. The acute response to endotoxaemia involves improvements in the autonomic nervous function as well as activation of innate immune mechanisms (Chalmeh et al., 2014). Measuring HR has been suggested to provide insight into the acute effect of sympathetic and cholinergic anti‐inflammatory pathways. In the current research, HRs increased substantially after the induction of endotoxaemia. Nonetheless, at hours 4.5 and 6 after endotoxin infusion, this parameter was significantly lower in the UFH 400 group than that in the UFH 200 ewes (Table 3; *p* < 0.05). The decreasing trend of HR in this group might be linked to lowering APR and balancing the autonomic nervous system.

Endotoxin has a significant impact on the structure and function of the lungs. Endotoxin causes both noticeable and subtle effects on the workings of the airways and the pulmonary circulation. These side‐effects include diffuse lung inflammation and pulmonary vascular endothelial damage. Endotoxin can also cause endothelial cell damage in vitro (Rojas et al., 2013). In this study, RR increased significantly during endotoxaemia and decreased following the therapeutic regimens. The lowest RR was observed in the UFH 400 at hours 4.5 and 6 following endotoxaemia induction (Table 3; *p* < 0.05), indicating that UFH has anti‐inflammatory effects on endotoxaemia in sheep.

Heparin‐induced thrombocytopenia (HIT) may occur following heparin administration, which can be fatal if left untreated. Platelet depletion following thrombocytopenia may be rapid or delayed, but platelet counts usually increase after discontinuation of heparin. Therefore, the decision to re‐administer heparin should be accompanied by platelet counting and monitoring (Ahmed et al., 2007). There are few studies on HIT in sheep (Connell et al., 2006). The sheep in this study had not previously received UFH and they did not receive UFH again after treatment in this research, therefore, they were less likely to develop complications from HIT. Platelet count was not performed in this study but clinical observations after this experiment did not show any signs of HIT in these sheep. Based on the present study, it can be stated that according to the method used in this research, UFH can be used due to its anti‐inflammatory properties in endotoxic sheep.

## 5 CONCLUSIONS

In conclusions, we shed light on the fact that UFH acts as an anti‐inflammatory mediator in sheep by decreasing inflammatory cytokines and acute phase proteins, modulating oxidative stress biomarkers and lowering MOD after *E. coli* serotype O55:B5‐induced endotoxaemia. Furthermore, the anti‐inflammatory effects of UFH at 400 IU/kg were substantially higher than those of UFH at 200 IU/kg; this exhibited the dose–dependent action of this drug. The current study found that UFH has therapeutic effects in the treatment of Ovine endotoxaemia as an inflammatory model. It is possible that the findings be applied to the treatment of other inflammatory conditions and further studies are needed. This research examined the effect of two doses of UFH and higher doses may have more anti‐inflammatory effects that require further studies.

## CONFLICT OF INTEREST

The authors declare no conflict of interest.

## ANIMAL ETHICS

The present experimental study was conducted after being approved by the Iranian laboratory animal ethics framework under the supervision of the Iranian Society for the Prevention of Cruelty to Animals and Shiraz University Research Council (IACUC no: 4687/63).

## AUTHOR CONRIBUTIONS

Hesam Ashareyoun: Investigation, Methodology, Project administration, Resources, Writing original draft. Aliasghar Chalmeh: Conceptualization, Methodology, Formal analysis, Investigation, Resources, Data curation, Writing original draft, Writing – review & editing, Supervision, Project administration. Mehrdad Pourjafar: Validation, Resources, Writing – review & editing, Supervision, Project administration.

## References

[vms3720-bib-0001] Ahmed, I. , Majeed, A. , & Powell, R. (2007). Heparin induced thrombocytopenia: diagnosis and management update. Postgraduate Medical Journal, 83(983), 575–582. 17823223 10.1136/pgmj.2007.059188 PMC2600013

[vms3720-bib-0002] Aldous, S. J. (2013). Cardiac biomarkers in acute myocardial infarction. International Journal of Cardiology, 164, 282–294. 22341694 10.1016/j.ijcard.2012.01.081

[vms3720-bib-0003] Anderson, R. , Tintinger, G. , Cockeran, R. , Potjo, M. &, Feldman, C. (2010). Beneficial and harmful interactions of antibiotics with microbial pathogens and the host innate immune system. Pharmaceuticals, 3, 1694–1710. 27713324 10.3390/ph3051694 PMC4034004

[vms3720-bib-0004] Centanni, E. (1984). Untersuchungen über das infections‐fieber. Dtsch Tierarztl Wochenschr, 20, 148–179.

[vms3720-bib-0005] Chalmeh, A. , Badiei, K. , Pourjafar, M. & Nazifi, S. (2013). Acute phase response in experimentally *Escherichia coli* serotype O55:B5 induced endotoxemia and its comparative treatment with dexamethasone and flunixin meglumine in Iranian fat‐tailed sheep. Veterinarski Arhiv, 83, 301–312. https://hrcak.srce.hr/101904

[vms3720-bib-0006] Chalmeh, A. , Badiei, K. , Pourjafar, M. , Nazifi, S. , Heidari, S. M. M. , Heidari, M. & Babazadeh, M. (2014). Alterations in electrocardiographic parameters and serum cardiac biomarkers in an ovine experimental endotoxemia model. Journal of Faculty of Veterinary Medicine, Istanbul University, 40, 211–219.

[vms3720-bib-0007] Christoffersen, M. , Baagoe, C. D. , Jacobsen, S. , Bojesen, A. M. , Petersen, M. R. & Lehn‐Jensen, H. (2010). Evaluation of the systemic acute phase response and endometrial gene expression of serum amyloid A and pro‐ and anti‐inflammatory cytokines in mares with experimentally induced endometritis. Veterinary Immunology and Immunopathology, 138, 95–105. 20728224 10.1016/j.vetimm.2010.07.011

[vms3720-bib-0008] Connell, J. M. , Khalapyan, T. , Al‐Mondhiry, H. , Rosenberg, G. , & Weiss, W. J. (2006). Anticoagulation of juvenile sheep and goats with heparin, warfarin, and clopidogrel. ASAIO Journal, 52(2), 29A. 10.1097/MAT.0b013e31802e192b 17413565

[vms3720-bib-0009] Constable, P. D. , Hinchcliff, K. W. , Done, S. H. & Gruenberg, W. (2017). Veterinary medicine: A textbook of the diseases of cattle, horses, sheep, pigs and goats (pp. 59–67). 11th ed. Elsevier;

[vms3720-bib-0010] Coskun, D. , Corum, O. & Yazar, E. (2020). Effect of supportive therapy on the pharmacokinetics of intravenous marbofloxacin in endotoxemic sheep. Journal of Veterinary Pharmacology and Therapeutics, 43, 288–296. 32133667 10.1111/jvp.12849

[vms3720-bib-0011] Derhaschnig, U. , Pernerstorfer, T. , Knechtelsdorfer, M. , Hollenstein, U. , Panzer, S. & Jilma, B. (2003). Evaluation of anti‐inflammatory and anti‐adhesive effects of heparins in human endotoxemia. Critical Care Medicine, 31, 1108–1112. 12682480 10.1097/01.CCM.0000059441.70680.DC

[vms3720-bib-0012] Eckersall, P. D. & Bell, R. (2010). Acute phase proteins: biomarkers of infection and inflammation in veterinary medicine. Veterinary Journal, 185, 23–27. 20621712 10.1016/j.tvjl.2010.04.009

[vms3720-bib-0013] Erel, O. (2004). A novel automated direct measurement method for total antioxidant capacity using a new generation, more stable ABTS radical cation. Clinical Biochemistry, 37, 277–285. 15003729 10.1016/j.clinbiochem.2003.11.015

[vms3720-bib-0014] Famularo, G. , De Simone, C. & Nicotra, G. C. (2003). Jaundice and the sepsis syndrome: a neglected link. European Journal of Internal Medicine, 14, 269–271. 12919846 10.1016/s0953-6205(03)00072-4

[vms3720-bib-0015] Haneberg, B. , Gutteberg, T. J. , Moe, P. J. , Osterud, B. , Bjorvatn, B. & Lehmann, E. H. (1983). Heparin for infants and children with meningococcal septicemia. NIPH Annals, 6, 43–47. 6353278

[vms3720-bib-0016] Jaimes, F. , De La Rosa, G. , Arango, C. , Fortich, F. , Morales, C. , Aguirre, D. & Patiño, P. (2006). A randomized clinical trial of unfractioned heparin for treatment of sepsis (the HETRASE study): design and rationale [NCT00100308]. Trials, 7, 19. 16729879 10.1186/1745-6215-7-19 PMC1482716

[vms3720-bib-0017] Kobashi, H. , Toshimori, J. & Yamamoto, K. (2013). Sepsis‐associated liver injury: incidence, classification and the clinical significance. Hepatology Research, 43, 255–266. 22971102 10.1111/j.1872-034X.2012.01069.x

[vms3720-bib-0018] Li, L. F. , Huang, C. C. , Lin, H. C. , Tsai, Y. H. , Quinn, D. A. & Liao, S. K. (2009). Unfractionated heparin and enoxaparin reduce high‐stretch ventilation augmented lung injury: a prospective, controlled animal experiment. Critical Care, 13, R108. 19580651 10.1186/cc7949 PMC2750150

[vms3720-bib-0019] Li, X. , Li, L. , Shi, Y. , Yu, S. & Ma, X. (2020). Different signaling pathways involved in the anti‐inflammatory effects of unfractionated heparin on lipopolysaccharide‐stimulated human endothelial cells. Journal of Inflammation, 17, 5. 32063752 10.1186/s12950-020-0238-7 PMC7011532

[vms3720-bib-0020] Li, X. , Li, Z. , Zheng, Z. , Liu, Y. & Ma, X. (2013). Unfractionated heparin ameliorates lipopolysaccharide‐induced lung inflammation by downregulating nuclear factor‐κB signaling pathway. Inflammation, 36, 1201–1208. 23690274 10.1007/s10753-013-9656-5

[vms3720-bib-0021] Li, X. & Ma, X. (2017). The role of heparin in sepsis: much more than just an anticoagulant. British Journal of Hematology, 179, 389–398. 10.1111/bjh.14885 28832958

[vms3720-bib-0022] Lida, A. , Yoshidome, H. , Shida, T. , Kimura, F. , Shimizu, H. , Ohtsuka, M. , Morita, Y. , Takeuchi, D. & Miyazaki, M. (2009). Does prolonged biliary obstructive jaundice sensitize the liver to endotoxemia? Shock, 31, 397–403. 18665046 10.1097/SHK.0b013e31818349ea

[vms3720-bib-0023] Ludwig, R. G. (2009). Therapeutic use of heparin beyond anticoagulation. Current Drug Discovery Technologies, 6, 281–289. 20025596 10.2174/157016309789869001

[vms3720-bib-0024] Marshall, J. C. (2012). New translational research provides insights into liver dysfunction in sepsis. PLoS Medicine, 9, e1001341. 23152725 10.1371/journal.pmed.1001341 PMC3496662

[vms3720-bib-0025] Miranda, M. L. , Prota, L. F. M. , Silva, M. J. B. , Sicuro, F. L. , Furtado, E. S. , Santos, A. O. M. T. & Bouskela, E. (2014). Protective microcirculatory and anti‐inflammatory effects of heparin on endotoxemic hamsters. Medical Express, 1, 127–134.

[vms3720-bib-0026] Mousavi, S. , Moradi, M. , Khorshidahmad, T. & Motamedi, M. (2015). Anti‐inflammatory effects of heparin and its derivatives: a systematic review. Advances in Pharmacological Sciences, 2015, 1–14. 10.1155/2015/507151 PMC4443644 26064103

[vms3720-bib-0027] Munford, R. S. (2016). Endotoxemia‐menace, marker, or mistake? Journal of Leukocyte Biology, 100, 687–698. 27418356 10.1189/jlb.3RU0316-151R PMC5014740

[vms3720-bib-0028] Parameswaran, N. & Patial, S. (2010). Tumor necrosis factor‐α signaling in macrophages. Critical Reviews in Eukaryotic Gene Expression, 20, 87–103. 21133840 10.1615/critreveukargeneexpr.v20.i2.10 PMC3066460

[vms3720-bib-0029] Patel, J. J. , Taneja, A. , Niccum, D. , Kumar, G. , Jacobs, E. & Nanchal, R. (2013). The association of serum bilirubin levels on the outcomes of severe sepsis. Journal of Intensive Care Medicine, 30, 23–29. 23753252 10.1177/0885066613488739

[vms3720-bib-0030] Peek, S. F. , Apple, F. S. , Murakami, M. A. , Crump, P. M. & Semrad, S. D. (2008). Cardiac isoenzymes in healthy Holstein calves and calves with experimentally induced endotoxemia. Canadian Journal of Veterinary Research, 72, 356–361. 18783025 PMC2442679

[vms3720-bib-0031] Poterucha, T. J. , Libby, P. & Goldhaber, S. Z. (2017). More than an anticoagulant: do heparins have direct anti‐inflammatory effects? Thrombosis and Haemostasis, 117, 437–444. 27975101 10.1160/TH16-08-0620

[vms3720-bib-0032] Pourjafar, M. , Chalmeh, A. , Badiei, K. , Nazifi, S. , Keshavarz, S. & Naghib, M. (2013). Correlations among homocysteine, cardiac troponin I and cardiac enzymes in different ages of clinically healthy male dromedary camels. Iranian Journal of Veterinary Medicine, 7, 201–206.

[vms3720-bib-0033] Redmond, J. M. , Gillinov, A. M. , Stuart, R. S. , Zehr, K. J. , Winkelestein, J. A. , Herskowitz, A. , Cameron, D. E. & Baumgartner, W. A. (1993). Heparin‐coated bypass circuits reduce pulmonary injury. Annals of Thoracic Surgery, 56, 474–479. 8104392 10.1016/0003-4975(93)90882-i

[vms3720-bib-0034] Rivera, C. A. , Tcharmtchi, M. H. , Mendoza, L. & Smith, C. W. (2003). Endotoxemia and hepatic injury in a rodent model of hind limb unloading. Journal of Applied Physiology, 95, 1656–1663. 12794033 10.1152/japplphysiol.00302.2003

[vms3720-bib-0035] Rodrigues, L. O. C. P. , Graça, R. S. F. & Carneiro, L. A. M. (2018). Integrated stress responses to bacterial pathogenesis patterns. Frontiers in Immunology, 9, 1306. 29930559 10.3389/fimmu.2018.01306 PMC5999787

[vms3720-bib-0036] Rojas, M. , Parker, R. E. , Thorn, N. , Corredor, C. , Iyer, S. S. , Bueno, M. , Mroz, L. , Cardenes, N. , Mora, A. L. , Stecenko, A. A. & Brigham, K. L. (2013). Infusion of freshly isolated autologous bone marrow derived mononuclear cells prevents endotoxin‐induced lung injury *in* an *ex‐vivo* perfused swine model. Stem Cell Research and Therapy, 4, 26. 23497755 10.1186/scrt174 PMC3706906

[vms3720-bib-0037] Sack, H. G. (2018). Serum amyloid A: a review. Molecular Medicine, 24, 46. 30165816 10.1186/s10020-018-0047-0 PMC6117975

[vms3720-bib-0038] Schiffer, E. R. , Reber, G. , Moerloose, P. & Morel, D. R. (2002). Evaluation of unfractioned heparin and recombinant hirudin on survival in a sustained ovine endotoxin shock model. Critical Care Medicine, 30, 2689–2699. https://pubmed.ncbi.nlm.nih.gov/12483060/ 12483060 10.1097/00003246-200212000-00013

[vms3720-bib-0039] Schroder, K. , Hertzog, P. J. , Ravasi, T. & Hume, D. A. (2004). Interferon‐γ: an overview of signals, mechanisms and functions. Journal of Leukocyte Biology, 75, 163–189. 14525967 10.1189/jlb.0603252

[vms3720-bib-0040] Svenmarker, S. , Sandstr¨om, E. , Karlsson, T. , Jansson, E. , Häggmark, S. , Lindholm, R. , Appleblad, M. & Aberg, T. (1997). Clinical effects of the heparin coated surface in cardiopulmonary bypass. European Journal of Cardio‐Thoracic Surgery, 11, 957–964. 9196315 10.1016/s1010-7940(96)01132-3

[vms3720-bib-0041] Tsikas, D. (2017). Assessment of lipid peroxidation by measuring malondialdehyde (MDA) and relatives in biological samples: analytical and biological challenges. Analytical Biochemistry, 524, 13–30. 27789233 10.1016/j.ab.2016.10.021

[vms3720-bib-0042] Uchino, S. (2010). Creatinine. Current Opinion in Critical Care, 16, 562–567. 20736825 10.1097/MCC.0b013e32833ea7f3

[vms3720-bib-0043] Valko, M. , Leibfritz, D. , Moncol, J. , Cronin, M. T. D. , Mazur, M. & Telser, J. (2007). Free radicals and antioxidants in normal physiological functions and human disease. International Journal of Biochemistry and Cell Biology, 39, 44–84. 16978905 10.1016/j.biocel.2006.07.001

[vms3720-bib-0044] Wang, H. , Liu, H. , Jia, Z. , Olsen, C. , Litwin, S. , Guan, G. & Yang, T. (2010). Nitro‐oleic acid protects against endotoxin‐induced endotoxemia and multiorgan injury in mice. American Journal of Physiology‐Renal Physiology, 298, 754–762. 10.1152/ajprenal.00439.2009 PMC2838591 20032118

[vms3720-bib-0045] Wyns, H. , Plessers, E. , De Backer, P. , Meyer, E. & Croubels, S. (2015). *In vivo* porcine lipopolysaccharide inflammation models to study immunomodulation of drugs. Veterinary Immunology and Immunopathology, 166, 58–69. 26099806 10.1016/j.vetimm.2015.06.001

[vms3720-bib-0046] Yao, Y. , Wang, D. & Yin, Y. (2013). Advances in sepsis‐associated liver dysfunction. Burns and Trauma, 2, 97. 10.4103/2321-3868.132689 PMC5012093 27602369

[vms3720-bib-0047] Zhao, D. , Ding, R. , Liu, Y. , Yin, X. , Zhang, Z. & Ma, X. (2017). Unfractionated heparin protects the protein C system against lipopolysaccharide‐induced damage *in vivo* and *in vitro* . Experimental and Therapeutic Medicine, 14, 5515–5522. 29285085 10.3892/etm.2017.5236 PMC5740512

